# Correction: Identification and validation of a m7G-related lncRNA signature for predicting the prognosis and therapy response in hepatocellular carcinoma

**DOI:** 10.1371/journal.pone.0331772

**Published:** 2025-09-05

**Authors:** Yue-Ling Peng, Ya-Fang Dong, Li-Li Guo, Mu-Ye Li, Hui Liao, Rong-Shan Li

There is an error in affiliation 1 for authors Yue-Ling Peng and Rong-Shan Li. The correct affiliation 1 is: Department of Nephrology, Fifth Hospital of Shanxi Medical University (Shanxi Provincial People’s Hospital).
